# Interaction Effects Between Low Self-Control and Meaning in Life on Internet Gaming Disorder Symptoms and Functioning in Chinese Adolescents: Cross-Sectional Latent Moderated Structural Equation Modeling Study

**DOI:** 10.2196/59490

**Published:** 2024-11-04

**Authors:** Ted CT Fong, Kunjie Cui, Paul SF Yip

**Affiliations:** 1 Research Hub of Population Studies The University of Hong Kong Pokfulam Hong Kong China (Hong Kong); 2 Centre on Behavioral Health The University of Hong Kong Pokfulam Hong Kong China (Hong Kong); 3 Research Institute of Social Development Southwestern University of Finance and Economics Chengdu China; 4 The HKJC Centre for Suicide Research and Prevention The University of Hong Kong Pokfulam Hong Kong China (Hong Kong); 5 Department of Social Work and Social Administration The University of Hong Kong Pokfulam Hong Kong China (Hong Kong)

**Keywords:** Chinese, impulsivity, interaction effects, internet gaming disorder, latent moderation, meaning in life, self-control, temper

## Abstract

**Background:**

Internet gaming disorder (IGD) is an emerging behavioral addiction with mental health implications among adolescents. Low self-control is an established risk factor of IGD. Few studies have, however, examined the moderating role of meaning in life (MIL) on the relationships between low self-control and IGD symptoms and functioning.

**Objective:**

This study aimed to examine the effects of low self-control and MIL and their interaction effects on IGD symptoms and family and school functioning in a structural equation model.

**Methods:**

A sample of 2064 adolescents (967, 46.9% male; mean age 14.6 years) was recruited by multistage cluster random sampling from 5 middle schools in Sichuan, China, in 2022. The participants completed a self-report questionnaire with validated measures on low self-control, presence of MIL, search for MIL, IGD symptoms, school commitment, and family functioning. Construct validity, measurement invariance, and structural invariance of the measures were evaluated by confirmatory factor analysis across sex. Structural equation modeling was conducted to examine the indirect effects of low self-control and MIL on family and school functioning through IGD symptoms. Latent moderated structural equation modeling was performed to examine the interaction effects between low self-control and MIL on IGD symptoms, school commitment, and family functioning.

**Results:**

All scales showed satisfactory model fit and scalar measurement invariance by sex. Males showed significantly greater IGD symptoms and lower levels of self-control (Cohen *d*=0.25-1.20, *P*<.001) than females. IGD symptoms were significantly and positively associated with impulsivity (β=.20, *P*=.01), temper (β=.25, *P*<.001), and search for meaning (β=.11, *P*=.048) and significantly and negatively associated with presence of meaning (β=–.21, *P*<.001). Presence of MIL and impulsivity showed a significant and negative interaction effect (β=–.11, SE .05; *P=*.03) on IGD symptoms. The positive effect of impulsivity on IGD symptoms was stronger among adolescents with low presence of MIL than those with high presence of MIL. Temper showed significant and positive interaction effects with presence of MIL (β=.08, SE .04; *P=*.03) and search for MIL (β=.08, SE .04; *P=*.04) on family functioning. The negative effects of temper on family functioning were stronger among adolescents with low levels of MIL than among those with high levels of MIL.

**Conclusions:**

This study provides the first findings on the interaction effects between low self-control and presence of MIL and search for MIL on IGD symptoms and functioning among a large sample of adolescents in rural China. The results have implications for targeted interventions to help male adolescents with lower self-control and presence of meaning.

## Introduction

Technological advances in the past decade have contributed to increased gaming behaviors among younger generations. Internet gaming disorder (IGD) is a psychiatric condition involving persistent internet gaming behaviors that lead to significant distress [1]. IGD refers to the frequent occurrence of at least 5 of the 9 diagnostic criteria of *DSM-5* (*Diagnostic and Statistical Manual of Mental Disorders* [Fifth Edition]) such as preoccupation (obsession with gaming), withdrawal symptoms, tolerance (need to play internet games for longer times or with greater intensity), displacement (neglect of work, study, or social life due to gaming), and deception (tendency to hide gaming time or negative consequences) [2]. Though a previous study [3] found only a minimal prevalence (0.3%-1%) of gamers qualified for IGD, this phenomenon has been aggravated by the social isolation brought on by the COVID-19 pandemic [4]. A recent meta-analysis estimated the pooled prevalence of IGD to be 8.8% among adolescents [5].

Adolescence is a crucial transition period with greater vulnerability to addictive behaviors and adolescents exhibit higher tendencies toward sensation-seeking and risk-taking behaviors [6]. Apart from existing risk factors of IGD such as loneliness, hopelessness, being bullied, and low self-esteem [5,7], low self-control has been positively linked with maladaptive gaming motivations and IGD symptoms in adolescents [8-10]. Impulsivity and temper have shown significant associations with greater IGD symptoms in adolescent samples [11,12]. IGD has been associated with poor family functioning and school achievement in adolescents [13,14]. Recent studies have linked IGD symptoms with parental factors and family conflicts [15,16].

Adolescents may play internet games for motivations such as socialization, risk-seeking, achievement, immersion, or escape from reality [17,18]. The self-determination theory posits that adolescents seek to fulfil 3 basic psychological needs: autonomy, competence, and relatedness [19]. In the gaming context, adolescents could make decisions and control their virtual characters to acquire a sense of autonomy in the context of “virtual reality.” They could gain a sense of competence by overcoming in-game challenges and tasks and achieve a sense of relatedness by connecting with other gamers. A previous study [8] found that failure to meet these psychological needs could undermine individuals’ self-control in regulating behaviors and impulses, which in turn contributes to greater IGD symptoms.

Meaning in life (MIL) measures the coherence and purpose of one’s life [20] and is operationalized in 2 dimensions: presence of MIL and search for MIL [21]. Though a recent study [22] found that MIL mediated the relationship between basic psychological needs and IGD, the authors only used the total MIL score and did not differentiate between the 2 dimensions. Lower presence of MIL has been consistently associated with greater IGD symptoms in adolescents [23-25]. In comparison, search for MIL has shown positive or nonsignificant associations with IGD symptoms [24,25]. A previous study found that the identity formation process during adolescence could contribute to internet gaming behaviors in the search for identity [26]. Excessive internet gaming could make this exploration maladaptive and lead to negative player identity [27]. The paradoxical roles of the 2 MIL factors on IGD symptoms warrant further investigation.

Both self-control and the presence of MIL could be important psychological resources for resilience and coping. Despite the negative association between search for MIL and subjective well-being [28], the presence of MIL showed positive moderating effects on happiness when individuals were searching for MIL. Schnell and Krampe [29] found that the presence of MIL could buffer the adverse effect of low self-control on mental distress. Another study [30] found that presence of MIL and search for MIL attenuated and amplified the effects of bullying victimization on IGD, respectively, in Chinese male adolescents. However, no existing studies have investigated the interaction effects between MIL and low self-control on IGD symptoms and functioning. Such an investigation would provide insights into how adolescents’ presence of MIL and search for MIL may moderate the effects of low self-control on IGD symptoms and functioning.

**Figure 1:**
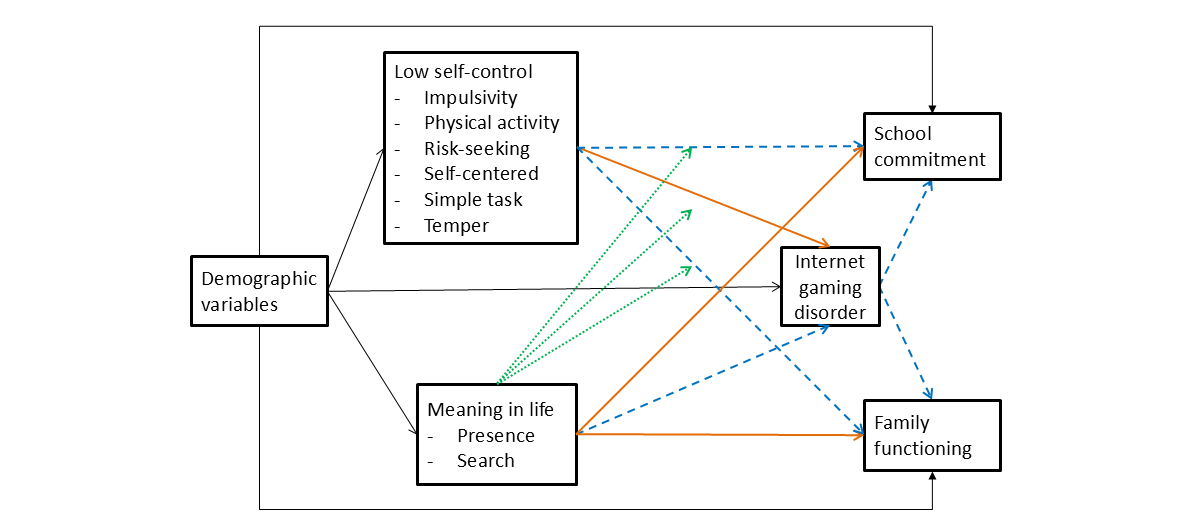
Conceptual model and hypothesized relationships among the study variables in the present study. Positive and negative associations are hypothesized and depicted in solid orange arrows and dash blue arrows, respectively. The dotted green arrows denote the potential moderating role of meaning in life on the relationships between low self-control and outcome variables.

This study had 3 objectives. Figure 1 depicts the conceptual model and hypothesized relationships of the present study. In the figure, positive and negative associations are hypothesized and depicted in orange solid arrows and blue dashed arrows, respectively. The green dotted arrows denote the potential moderating role of meaning in life on the relationships between low self-control and outcome variables. First, we aimed to examine the relationships among low self-control, MIL, IGD symptoms, and family and school functioning in a structural equation model (SEM). Second, we investigated the indirect effects of low self-control and MIL on adolescents’ family and school functioning through IGD symptoms. Third, we aimed to examine the interactive effects between MIL and low self-control on IGD symptoms and family and school functioning of adolescents. The study proposed several hypotheses. In hypothesis 1, low self-control would be positively associated with IGD symptoms and negatively with family and school functioning. In hypothesis 2, the presence of MIL would be negatively associated with IGD symptoms. In hypothesis 3, search for MIL would show positive or nonsignificant associations with IGD symptoms. In hypothesis 4, presence of MIL and search for MIL would have interaction effects with low self-control on IGD symptoms and family and school functioning.

## Methods

### Study Design and Procedure

This study was cross-sectional in nature and adopted a multistage cluster random sampling method. In the first stage, 5 middle schools were randomly sampled in Liangshan Yi Autonomous prefecture of Sichuan province in China. The Liangshan prefecture is an impoverished region known with its high ethnic diversity in rural China [31]. Adolescents in this region are at risks of developing IGD given the shortage of qualified teachers, limited collaboration between schools and families, and low effectiveness of family education [10]. Next, grade 7-8 classes were randomly selected from each school between June and July 2022. A total of 2378 students were invited from 38 classes through invitation letters in the 5 middle schools. Inclusion criteria of the study were age 11 to 17 years, students studying in grade 7 or 8, and ability to read simplified Chinese. Exclusion criteria of the study were presence of substantial visual and psychiatric impairment that interfered with completion of the questionnaire and inability to obtain written informed consent from the parent or guardian of the students. The eligibility of the students to participate was screened by a research assistant with reference to school records. The overall response rate of the survey was 86.8% and 2064 participants voluntarily elected to join the survey.

### Measures

In this study, the self-report questionnaire included the following measures on low self-control, MIL, IGD symptoms, school commitment, and family functioning. The questionnaire also asked the following sociodemographic characteristics of the participants, namely, sex, age, ethnicity, urban or rural registration, and whether they were left-behind children. The questionnaire was administered in a paper-and-pencil format and completed in 20-30 minutes in classroom settings without interference from the school teachers. Participants were reminded to avoid discussion during the survey completion and encouraged to answer the questionnaire honestly. The questionnaire was field tested in May 2022 with minor revisions based on comments from the adolescents.

Low self-control was measured by the Grasmick Low Self-Control Scale [32]. This 23-item scale assessed 6 trait types of low self-control: impulsivity (3 items), physical activity (4 items), risk-seeking (4 items), self-centeredness (4 items), simple task (4 items), and temper (4 items). The items are scored on a 4-point scale ranging from 1=“strongly disagree” to 4=“strongly agree.” Higher scores indicated lower levels of self-control.

MIL was assessed by the 6-item Meaning in Life Questionnaire–short form [21] on 2 dimensions of MIL: presence of MIL (3 items) and search for MIL (3 items). Items are scored on a 7-point scale ranging from 1=“absolutely untrue” to 7=“absolutely true.” Higher scores indicated higher levels of presence of and search for MIL.

The 9-item IGD Scale–short form [33] was used to assess the severity of IGD symptoms according to the *DSM-5* diagnostic criteria over the past year. The 9 items are scored on a 5-point scale ranging from 1=“Never” to 5=“Very often” and added up for the total IGD score (theoretical range=9-45). Higher scores indicated greater levels of IGD symptoms.

School commitment was assessed by 4 self-constructed items on a 5-point scale from 1=“strongly disagree” to 5=“strongly agree.” Example of items included “Getting good grades is important to me.” Family functioning was measured by 5 self-constructed items on a 5-point scale ranging from 1=“absolutely untrue” to 5=“absolutely true.” Example of items included “When problems arise, we are able to work together to solve them and rely on each other.” Higher scores indicated greater levels of commitment to school and family functioning.

Composite reliability of the latent factors was evaluated using McDonald omega (ω) with values ≥.70 indicating good reliability. All of the measures showed good levels of reliability (ω=.71-.94).

### Sample Size Planning

Power analysis was performed through Monte Carlo simulation with 1000 replications to determine the required sample size for SEM [34]. In the SEM, we specified a latent predictor with 4 observed indicators, a latent mediator with 3 observed indicators, and a latent outcome variable with 5 observed indicators. The factor loadings (λ) of the latent factors were fixed at 0.7. The acceptable α error level was set at *P*<.05. Effect size of the standardized path coefficients was denoted by 0.14 (small effect) and 0.39 (moderate effect) [35]. The sample size of male (n=967) and female (n=1097) subsamples showed adequate statistical power for detecting statistical significance for direct effects (91.2%-97.3%) and indirect effects (83.5%-89.6%) with small effect sizes.

### Data Analyses

#### Construct Validity of Measures

This study conducted data analysis in 4 steps. The first step examined the factorial validity of the measures by confirmatory factor analysis (CFA). The 47 items on low self-control, MIL, IGD symptoms, school commitment, and family functioning were analyzed in separate CFA models and a combined 11-factor CFA model in Mplus (version 8.6, Muthén and Muthén) [36]. Preliminary item analysis found substantial floor effects for the low self-control and IGD items and substantial ceiling effects for the school commitment and family functioning items. Given the skewed distributions of the items, these items were treated as ordinal items using the robust weighted least square estimator [37]. Model fit was evaluated through the following criteria on the fit indices [38]: comparative fit index (CFI) ≥.95, Tucker-Lewis index (TLI) ≥.95, root-mean-square error of approximation (RMSEA) ≤.06, and standardized root-mean-square residuals (SRMR) ≤.06. Statistical significance was set at *P*<.05 in the present study.

#### Measurement and Structural Invariance Across Sex

The second step evaluated the measurement invariance of the scales across sex. Multiple group CFA estimated the configural invariance model with different item loadings and thresholds across sex. Scalar invariance model fixed all item loadings and thresholds to be equal across sex [39]. Model comparison was performed using the chi-square difference test and change in model fit indices, with ΔCFI >–.01 and ΔRMSEA and ΔSRMR <.01 supporting the more constrained model [40]. Structural invariance tests compared the means of latent factors across sex on a standardized metric. Cohen *d* denoted the effect sizes with cutoff scores of 0.2, 0.5, and 0.8 for small, moderate, and large sizes, respectively [41]. Missing data were minimal and were handled using full information maximum likelihood under the missing-at-random assumption [42].

#### Structural Equation Model

Third, SEM was conducted to examine the direct and indirect effects from low self-control and MIL to school commitment and family functioning through IGD symptoms. In the SEM, the 6 low self-control factors and 2 MIL factors were the latent predictors; the IGD factor was the latent mediator; and the school commitment and family functioning factors were the latent outcomes. The model included sex, age, ethnic minority, urban registration, and left-behind children as control variables. Indirect effects were estimated using 5000 bootstrap draws and considered statistically significant if their 95% bootstrapped CI excluded 0. *R*^2^ values denoted the proportion of explained variance for the dependent variables. Sensitivity analysis was performed by conducting multigroup SEM across sex to examine potential sex differences.

#### Interaction Effects Between MIL and Low Self-Control

In the fourth step, we conducted latent interaction analysis by adding interaction terms between the latent MIL factors and low self-control factors to the SEM separately. The latent interaction SEM was estimated using the XWITH option with numerical integration in Mplus, which allowed us to examine whether MIL factors moderated the relationships between low self-control and IGD symptoms and latent outcomes. Standardized coefficients for the latent interaction terms were interpreted in terms of direction and statistical significance. Simple slopes analysis was conducted to probe the interaction effects by testing the conditional effects of low-self-control on IGD symptoms and functioning at mean, low (mean –1.5SD), and high (mean + 1.5SD) levels of MIL.

### Ethical Considerations

The study received ethical approval from the Ethics Committee of the Southwestern University of Finance and Economics (approval number 2422CSH068). The study objectives and procedures were clearly explained to the participants by the research assistant in each classroom. Participation was completely voluntary. Written informed consent was obtained from the parents or legal guardians of the adolescents. Participants had the right to opt out of the survey and not answer specific questions without any adverse outcomes. All collected data was anonymized to preserve confidentiality of personal information. No financial compensation was provided to the study participants. All procedures contributing to this work comply with the Helsinki Declaration of 1975, as revised in 2008.

## Results

### Sample Characteristics

As shown in Table S1 in Multimedia Appendix 1, a total of 1097 out of 2064 participants (53.1%) were females, and the average age was 14.6 (SD 1.10) years. Most of the participants were ethnic minorities (1931/2064, 93.6%) and had rural registration (1929/2064, 93.5%). Over 1 quarter (592/2064, 28.7%) of the sample were left-behind children in their hometown. Out of 2064, a total of 1281 (62.1%) participants were regular gamers who played internet games for at least 3 hours per week. Overall, the sample reported moderate levels of self-control, presence of MIL, and search for MIL, moderately high levels of school commitment and family functioning, and low levels of IGD symptoms (Multimedia Appendix 1). The sample prevalence of IGD was 2% (36/2064). The IGD prevalence was significantly higher (*χ*^2^_1_=14.1, *P*<.001) in males (prevalence=3%; 28/967) than females (prevalence=1%; 8/1097).

### Construct Validity of Measures

All measurement scales showed acceptable model fit to the data in CFA with CFI>.95, TLI≥.95, RMSEA≈.06, and SRMR<.06 (Multimedia Appendix 2). Substantial factor loadings were found for low self-control (λ=0.53-0.85), MIL (λ=0.68-0.87), IGD (λ=0.70-0.88), school commitment (λ=0.45-0.77), and family functioning (λ=0.83-0.93).

### Measurement and Structural Invariance Across Sex

Both configural and scalar invariance models provided adequate model fits across sex. Despite significant chi-square difference test between the 2 models (Δ*χ*^2^_155_=357.6, *P*<.001), the scalar invariance model did not show deterioration in fit indices. As Table 1 shows, males showed significantly higher latent means in physical activity, risk-seeking, self-centeredness, and IGD symptoms (*d*=0.25-1.20, *P*<.001) and significantly lower latent means in school commitment (*d*=–0.42, *P*<.001) than females. The latent mean differences in other factors were not significant (*d* =–0.08-0.14, *P*=.12-.86).

**Table 1:** Latent mean difference of males compared with females in the study variables in the scalar invariance model (N=2064).

Variables		Cohen *d* *^a,b^*	SE	*P* value
**Low self-control**				
	Impulsivity	0.03	0.054	.53
	Physical activity	0.33	0.056	<.001
	Risk-seeking	0.34	0.064	<.001
	Self-centeredness	0.25	0.062	<.001
	Simple task	0.14	0.057	.06
	Temper	0.01	0.053	.86
Presence of meaning		0.01	0.046	.77
Search for meaning		–0.07	0.048	.17
Internet gaming disorder symptoms		1.20	0.209	<.001
School commitment		–0.42	0.058	<.001
Family functioning		–0.08	0.051	.12

^a^All latent means were fixed at zeros in the female subgroup for identification purpose.

^b^Cohen *d*: SD for males over females.

### Structural Equation Model

**Figure 2:**
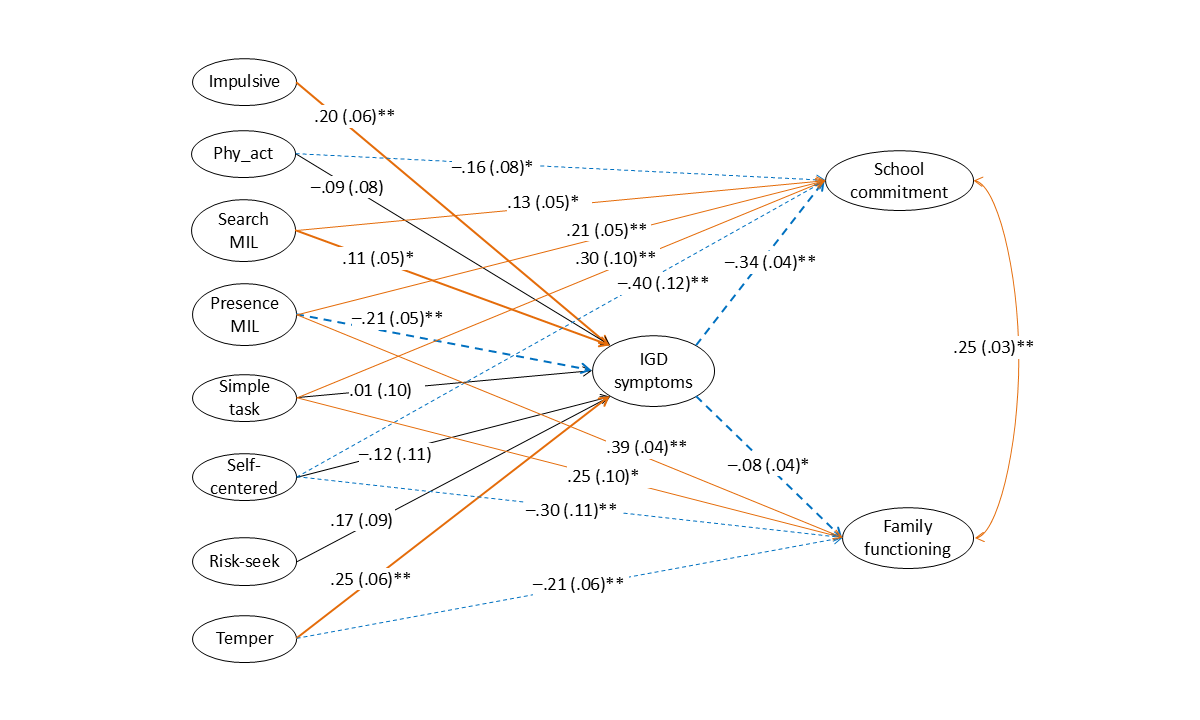
Standardized path coefficients and standard errors (in parenthesis) from low self-control factors and meaning in life factors to school commitment and family functioning through internet gaming disorder symptoms in the structural equation model. *P<.05; **P<.01.

The SEM with covariates provided an adequate fit to the whole sample. Multimedia Appendix 3 shows the standardized effects of demographic variables on the latent factors in the model. Figure 2 depicts the standardized regression paths from low self-control and MIL to school commitment and family functioning through IGD symptoms. For simplicity of presentation, the figure does not display the 47 observed items of the latent variables, demographic variables, and their associated pathways. Positive and negative paths are represented by continuous orange lines and dash blue lines, respectively. Significant paths involving IGD symptoms are highlighted in bold. Residual correlation between school commitment and family functioning is shown in black dashed arrows. IGD symptoms were significantly and positively associated with impulsivity (β=.20, *P*=.01), temper (β=.25, *P*<.001), and search for meaning (β=.11, *P*=.048), and significantly and negatively associated with presence of meaning (β=–.21, *P*<.001). IGD symptoms were significantly and negatively associated with school commitment (β=–.34, *P*<.001) and family functioning (β=–.08, *P*=.03). The model explained 37.1%, 41.1%, and 33.7% of the variances of IGD symptoms, school commitment, and family functioning, respectively.

As Table 2 shows, out of the 6 low self-control factors, impulsivity and temper showed significant and negative indirect effects on school commitment and family functioning through IGD symptoms. The presence of MIL had significant and positive indirect effects on school commitment and family functioning through IGD symptoms. Search for MIL showed a significant and negative indirect effect on school commitment through IGD symptoms. Multigroup SEM found comparable indirect effects from impulsivity, temper, and presence of MIL to school commitment and family functioning through IGD symptoms across sex. The negative indirect effect from search for meaning to school commitment through IGD symptoms was significant in males (αβ=–.059, 95% CI –0.115 to –0.008) but not in females (αβ=–.012, 95% CI –0.074 to 0.046).

**Table 2:** Standardized indirect effects from low self-control and meaning in life to school commitment and family functioning through internet gaming disorder symptoms in the structural equation model (N=2064).

Predictor variable	School commitment	Family functioning
	Estimate (95% CI)	Estimate (95% CI)
Impulsivity	–0.066 (–0.119 to –0.016)	–0.016 (–0.041 to –0.001)
Physical activity	0.029 (–0.018 to 0.083)	0.007 (–0.005 to 0.025)
Risk-seeking	–0.058 (–0.132 to 0.003)	–0.014 (–0.038 to 0.000)
Self-centered	0.039 (–0.038 to 0.119)	0.010 (–0.011 to 0.034)
Simple task	–0.004 (–0.073 to 0.049)	–0.001 (–0.021 to 0.012)
Temper	–0.086 (–0.136 to –0.044)	–0.021 (–0.046 to –0.004)
Presence of meaning in life	0.071 (0.036 to 0.107)	0.018 (0.002 to 0.035)
Search for meaning in life	–0.037 (–0.075 to –0.006)	–0.009 (–0.023 to 0.000)

### Interaction Effects Between MIL and Low Self-Control

**Figure 3:**
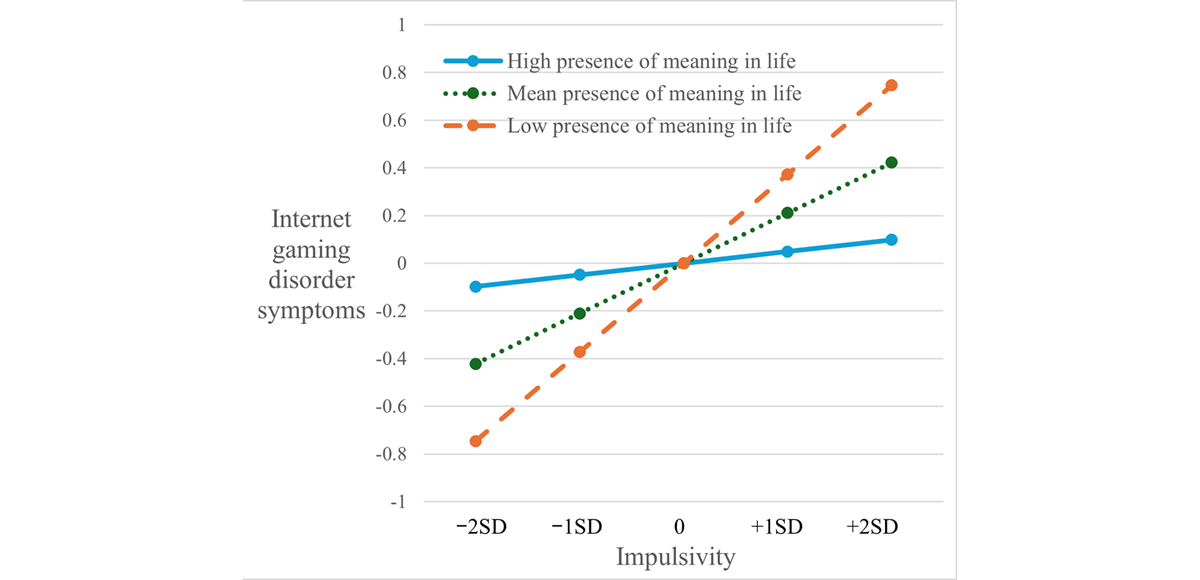
Effects of impulsivity on internet gaming disorder symptoms conditional on presence of meaning in life. For presence of meaning in life, high levels=mean+1.5SD and low levels=mean−1.5SD.

**Figure 4:**
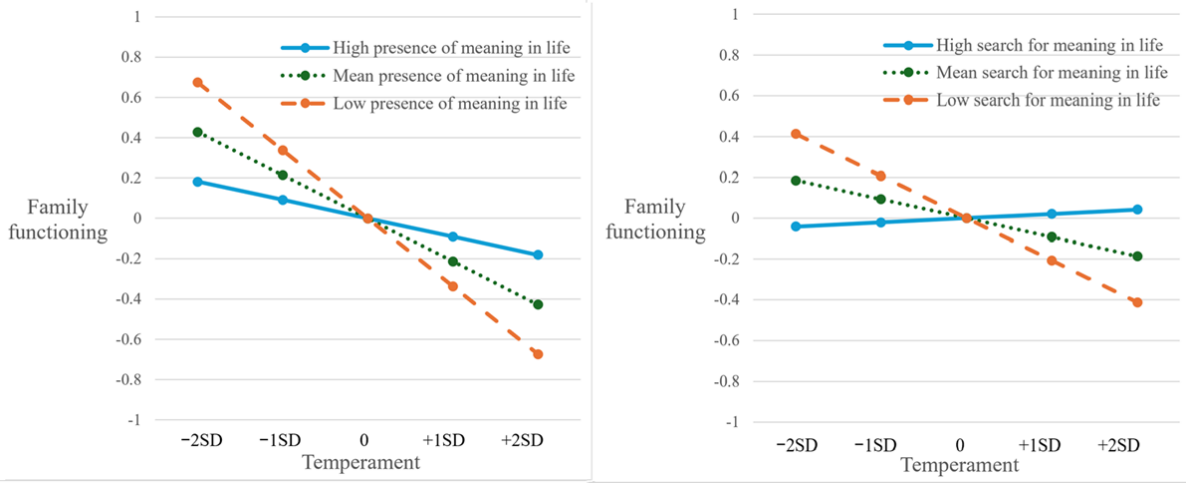
Effects of temper on family functioning conditional on presence of meaning in life and search for meaning in life. For presence of meaning in life and search for meaning in life, high levels=mean+1.5SD and low levels=mean−1.5SD.

Latent interaction SEM was conducted to examine 4 interaction effects between MIL factors and impulsivity and temper. Presence of MIL and impulsivity showed a significant and negative interaction effect (β=–.11, SE 0.05; *P=*.03) on IGD symptoms. As Figure 3 shows, the positive effect of impulsivity on IGD symptoms was stronger (dash orange line) among adolescents with low presence of MIL and was much weaker (solid blue line) among those with high presence of MIL. The effects of other 3 interaction terms on IGD symptoms were not significant (β=–.06-.01, SE 0.05-0.06; *P=*.17-.92). Both impulsivity and temper showed positive but nonsignificant interaction effects with presence of MIL and search for MIL on school commitment (β=.04-.08, SE 0.05; *P=*.09-.44). Presence of MIL and search for MIL showed significant and positive interaction effects (β=.08, SE 0.04; *P=*.03-.045) with temper on family functioning. As Figure 4 shows, the negative effects of temper on family functioning were stronger (dash orange lines) among adolescents with low levels of MIL and were much weaker (solid blue lines) among those with high levels of MIL.

## Discussion

### Principal Findings

This study evaluated the effects of low self-control and MIL on IGD symptoms and family and school functioning in a large sample of Chinese adolescents. Two low self-control factors (impulsivity and temper) were significantly associated with greater IGD symptoms, and 5 low self-control factors (except simple task) were directly or indirectly associated with worse school commitment and family functioning. These provided empirical support for hypothesis 1. IGD symptoms were found to have significant and negative associations with presence of MIL and significant and positive associations with search for MIL. These results are consistent with hypothesis 2 and hypothesis 3. Consistent with hypothesis 4, MIL factors showed interaction effects with impulsivity and temper on IGD symptoms and family functioning.

### Comparison With Previous Work

Consistent with previous literature [22-24,43], adolescents with greater presence of MIL showed lower IGD symptoms and better family functioning and school commitment. Presence of MIL has found a significant and negative indirect effect on suicidal ideation through psychological distress in Hong Kong young adults [44]. The indirect effects from low self-control and MIL to school commitment were stronger than those for family functioning. This appears to suggest greater relevance of IGD in the school context than the family context. Furthermore, this study found that presence of MIL could play a buffering role on the adverse effect of impulsivity on IGD symptoms among adolescents. This is consistent with previous findings on the moderating role of presence of MIL on drug use, suicide risk, and internet addiction [45-47].

In line with recent findings [25], search for MIL showed a positive effect on IGD symptoms after controlling for presence of MIL. In contrast to the positive direct effect from search for MIL to school commitment, the negative indirect effect through IGD symptoms implies that adolescents could search for MIL by internet gaming and maladaptive gaming behaviors could lead to decrement in school commitment. Nevertheless, search for MIL was found to positively modulate the negative effect of temper on family functioning. Overall, the present results suggest that search for MIL plays a complex role in relation to adolescents’ functioning through their IGD symptoms.

In our study, the sex differences in the IGD symptoms and self-control agree with the literature [8,48-50]. In a qualitative study [51], gaming was a meaningful activity for problem gamers and the gaming experience offered a sense of meaning to them. Our multigroup SEM results suggest a more salient role for search for MIL in predicting IGD symptoms and school commitment in males. A previous study [30] found that presence of MIL and search for MIL attenuated and amplified the effects of bullying victimization on IGD, respectively, in male adolescents but not in female adolescents. This discrepancy highlights greater service needs to foster a sense of meaning among male adolescents.

IGD is a prominent public health issue in Asian countries such as China and South Korea [52]. The present sample displayed substantially lower levels of IGD symptoms (Cohen *d*=0.87, *P*<.01) than that reported in a recent study among young people in Hong Kong [49]. Adolescents in China are only allowed to play internet games for at most 1.5 hours on weekdays and 3 hours on weekends and holidays [53]. It is plausible that the government restriction on internet gaming contributed to substantially lower severity of IGD in adolescents in China than in Hong Kong. However, presence of floor effects for low self-control and IGD and ceiling effects for functioning indicated potential lack of sensitivity of the measures in capture individual differences. Underestimation of the true variability could cast doubts on the validity of the present findings.

### Implications for Future Work

In Liangshan, many parents migrated to urban areas for work opportunities and better livelihood, resulting in many left-behind children. These adolescents showed lower levels of presence of meaning, family functioning, and self-control in the present sample. Previous studies have found inadequate parental supervision and greater loneliness among left-behind children in rural China [54,55]. Interestingly, ethnic minorities showed fewer IGD symptoms and higher school commitment than the Han counterparts. The high proportion of ethnic minorities denotes a sense of cultural similarity and better social support, both of which could contribute to lower IGD risks [56]. From a practical perspective, positive psychology interventions such as character strength intervention [57] and life skills development programs [58] could be implemented in schools to facilitate the adolescents’ search for identity and MIL to promote holistic development during adolescence. A previous study found that awareness of medicalization of IGD was linked with reduced gaming time, fewer maladaptive cognitions, and greater help-seeking intention in Chinese adolescents [59]. School-based prevention programs should aim to increase the adolescents’ awareness of the IGD symptoms and their adverse effects in a nonstigmatizing manner.

Besides, recent studies have suggested the use of mindfulness-based intervention [60] and self-control intervention [61] to reduce aggression and promote self-control in adolescents. For instance, a recent study [62] has examined the effectiveness of relapse prevention therapy on parent-child communication, parent-child relationships, and parenting needs for youths with IGD. A recent study identifies digital phenotypes (faster stroke acceleration, reduced word spacing, decreased deletion behavior, and longer horizontal strokes) by student tablet data for early detection of IGD among 168 Korean adolescents [63]. Large-scale studies are recommended to evaluate the effectiveness of digital phenotyping as an early detection method of IGD for adolescents. Further research should explore the associations between preferences toward gaming devices and genres and gamification user types [64]. The results could inform the development of targeted treatment programs toward different types of gamers in different gaming devices. Future studies could examine the inter-relationships between IGD and other risk factors such as social withdrawal, cybersex experiences, deviant behaviors at a symptom level through network analysis [65-67].

### Limitations

This study used the full latent variable approach to account for the measurement errors of the item indicators, which mitigates the measurement bias and provides more reliable estimates of the regression paths. However, the present study has a few limitations. First, the cross-sectional design did not permit inference of directional relationships between MIL, self-control, and IGD symptoms. Reciprocal effects from IGD to self-control and MIL could not be ruled out. Panel studies are needed to elucidate their temporal relationships. Second, the present study focused on the effects of low self-control and MIL on IGD symptoms and functioning. Omission of important confounding variables could lead to omitted variable bias in the present results. Further studies should examine the effects of factors such as maladaptive cognitions, peer influences, social withdrawal, resilience on IGD [68-70]. Third, the adolescents were recruited from 5 middle schools and were mostly ethnic minorities. There are self-selection and response biases associated with cluster sampling method. Cautions are warranted in generalizing the present results to the general population of adolescents in China.

Fourth, the present results were based solely on self-report data from the adolescents and were subject to common method bias. It was plausible that participants with IGD would under-report their symptoms, leading to an underestimation of the IGD severity in the sample. Future studies should incorporate additional sources of information from parents or teachers to provide a more accurate evaluation of IGD symptoms. Fifth, recent studies have indicated potential overlap between IGD and smartphone addiction and social media addiction [24,71,72]. A recent study found that gaming devices (computer or mobile phone) contributed to differences in gaming time, gaming motivations, and IGD symptoms [73]. It is plausible that IGD and smartphone addiction are simply manifestations of the same underlying disorder as a result of confounding effect of gaming device. Further studies should clarify the comorbid relationships and the nature of digital addictions through network analysis to ensure precise use of diagnostic categories.

### Conclusions

This study provided the first findings on the interaction effects between low self-control and presence of MIL and search for MIL on IGD symptoms and functioning among a large sample of adolescents in rural China. Presence of MIL and search for MIL could mitigate the adverse effects of impulsivity on IGD symptoms and temper on family functioning, respectively. The results have implications for targeted interventions to help male adolescents with lower self-control and presence of meaning.

## Appendix

Multimedia Appendix 1 Demographics and descriptive statistics of the sample.

Multimedia Appendix 2 Fit indices of confirmatory factor analysis models of the measurement scales, measurement invariance models across gender, and structural equation modeling models in the whole sample and across gender subgroups.

Multimedia Appendix 3 Standardized effects of demographic variables on the latent variables in the structural equation model.

## Abbreviations

CFA: confirmatory factor analysis
CFI: comparative fit index
*DSM-5*: *Diagnostic and Statistical Manual of Mental Disorders* (Fifth Edition)
IGD: Internet gaming disorder
MIL: meaning in life
RMSEA: root-mean-square error of approximation
SEM: structural equation modeling
SRMR: standardized root-mean-square residuals
TLI: Tucker-Lewis index

## Data Availability

The raw data and Mplus syntax analyzed in this study are available from the first author upon reasonable request.
