# Supplementary material for: Interaction Effects Between Low Self-Control and Meaning in Life on Internet Gaming Disorder Symptoms and Functioning in Chinese Adolescents: Cross-Sectional Latent Moderated Structural Equation Modeling Study
Source: J Med Internet Res. 2024 Nov 4;26:e59490. doi: 10.2196/59490 (PMC11574502; doi:10.2196/59490)
Supplement: Multimedia Appendix 1 [file jmir_v26i1e59490_app1.docx]

| **Multimedia Appendix 1.** Demographics and descriptive statistics of the sample | | |
| --- | --- | --- |
| Categorical variable | Value | |
| Gender, male, n(%) | 967 (46.9%) | |
| Ethnic minority, n(%) | 1931 (93.6%) | |
| Urban registration, n(%) | 135 (6.5%) | |
| Left-behind children, n(%) | 592 (28.7%) | |
| Continuous variable | Range | Mean (SD) |
| Age in years, mean (SD) | 12 – 17 | 14.6 (1.10) |
| Low self-control |  |  |
| Impulsivity | 1 – 4 | 2.06 (0.68) |
| Physical activity | 1 – 4 | 2.08 (0.65) |
| Risk seeking | 1 – 4 | 1.77 (0.65) |
| Self-centered | 1 – 4 | 1.63 (0.65) |
| Simple task | 1 – 4 | 1.93 (0.67) |
| Temper | 1 – 4 | 1.91 (0.66) |
| Meaning in life |  |  |
| Presence | 1 – 7 | 4.43 (1.46) |
| Search | 1 – 7 | 4.34 (1.40) |
| IGD symptoms | 9 – 45 | 13.0 (5.50) |
| School commitment | 1 – 5 | 3.98 (0.77) |
| Family functioning | 1 – 5 | 4.00 (0.94) |
| N = 2,064; IGD = internet gaming disorder; higher scores indicate lower levels of self-control, better meaning in life, school commitment, and family functioning, and more severe IGD symptoms. | | |
