# Supplementary material for: Interaction Effects Between Low Self-Control and Meaning in Life on Internet Gaming Disorder Symptoms and Functioning in Chinese Adolescents: Cross-Sectional Latent Moderated Structural Equation Modeling Study
Source: J Med Internet Res. 2024 Nov 4;26:e59490. doi: 10.2196/59490 (PMC11574502; doi:10.2196/59490)
Supplement: Multimedia Appendix 2 [file jmir_v26i1e59490_app2.docx]

| **Multimedia Appendix 2.** Fit indices of CFA models of the measurement scales, measurement invariance models across gender, and SEM models in the whole sample and across gender subgroups | | | | | | |
| --- | --- | --- | --- | --- | --- | --- |
| Model | *χ^2^* | *df* | RMSEA | CFI | TLI | SRMR |
| **CFA** |  |  |  |  |  |  |
| 6-factor CFA on LSC | 1493.7 | 215 | .054 (.051–.056) | .957 | .949 | .033 |
| 2-factor CFA on MIL | 74.2 | 8 | .064 (.051–.077) | .975 | .954 | .027 |
| 1-factor CFA on IGD | 308.0 | 27 | .071 (.064–.078) | .982 | .976 | .031 |
| 2-factor CFA on SC and FF | 158.0 | 26 | .050 (.042–.057) | .996 | .995 | .018 |
| Combined 11-factor CFA | 3565.7 | 979 | .036 (.035–.037) | .963 | .959 | .040 |
| **Invariance across gender** |  |  |  |  |  |  |
| Configural invariance | 4627.6 | 1958 | .036 (.035–.038) | .965 | .962 | .047 |
| Scalar invariance | 4842.6 | 2113 | .035 (.034–.037) | .965 | .964 | .047 |
| **SEM with covariates** |  |  |  |  |  |  |
| Whole sample | 3982.4 | 1159 | .034 (.033–.036) | .962 | .956 | .038 |
| Across gender subgroups | 5006.3 | 2377 | .033 (.031–.034) | .961 | .958 | .045 |
| CFA = confirmatory factor analysis; SEM = structural equation modeling; *χ^2^*= chi-square; df = degree of freedom; RMSEA = root mean square error of approximation; CFI = comparative fit index; TLI = Tucker-Lewis index; SRMR = standardized root mean square residuals; LSC = low self-control; MIL = meaning in life; IGD = internet gaming disorder; SC = school commitment; FF = family functioning. | | | | | | |
