# Supplementary material for: Interaction Effects Between Low Self-Control and Meaning in Life on Internet Gaming Disorder Symptoms and Functioning in Chinese Adolescents: Cross-Sectional Latent Moderated Structural Equation Modeling Study
Source: J Med Internet Res. 2024 Nov 4;26:e59490. doi: 10.2196/59490 (PMC11574502; doi:10.2196/59490)
Supplement: Multimedia Appendix 3 [file jmir_v26i1e59490_app3.docx]

| **Multimedia Appendix 3.** Standardized effects of demographic variables on the latent variables in the structural equation model | | | | | |
| --- | --- | --- | --- | --- | --- |
|  | Demographic variable | | | | |
| N = 2064 | Male | Age | Ethnic minority | Urban registration | Left–behind children |
| Latent variable | β (SE)^a^ | β (SE) | β (SE) | β (SE) | β (SE) |
| Low self-control: |  |  |  |  |  |
| Impulsivity | .03 (.03) | –.03 (.03) | –.03 (.03) | .05 (.03) | .05 (.03) |
| Physical activity | .18 (.03)^b^ | –.04 (.03) | –.04 (.03) | .03 (.03) | .04 (.03) |
| Risk-seeking | .16 (.03)^b^ | –.02 (.03) | –.01 (.03) | .05 (.03) | .05 (.02) |
| Self-centered | .14 (.03)^b^ | .02 (.03) | .01 (.03) | .07 (.03)^b^ | .06 (.02) |
| Simple task | .09 (.03)^b^ | .01 (.03) | –.05 (.03) | .05 (.03) | .07 (.03)^b^ |
| Temper | .04 (.03) | .02 (.03) | –.03 (.03) | .05 (.03) | .05 (.03) |
| Presence of meaning | .01 (.02) | –.08 (.02)^b^ | –.01 (.03) | –.01 (.03) | –.07 (.02)^b^ |
| Search for meaning | –.04 (.03) | –.07 (.03)^b^ | –.06 (.03) | –.01 (.03) | .01 (.03) |
| IGD symptoms | .41 (.02)^b^ | –.01 (.02) | –.08 (.03)^b^ | –.04 (.03) | –.01 (.02) |
| School commitment | –.05 (.03) | –.03 (.03) | .05 (.02) | –.05 (.03) | .04 (.02) |
| Family functioning | .01 (.03) | –.01 (.02) | .04 (.02) | –.02 (.02) | –.05 (.02) |
| ^a^β = standardized regression coefficients; SE = standard error; IGD = Internet gaming disorder.  ^b^*P* < .01. | | | | | |

Age was negatively associated with presence of MIL and search for MIL (*β* = -.07 – -.08, *P* = .001 – .004). Ethnic minority was negatively associated with search for meaning and IGD symptoms (*β* = -.06 – -.08, *P* = .001 – .01) and positively associated with school commitment (*β* = .05, *P* = .009). Urban registration was associated with higher levels of self-centeredness and simple task (β = .05 – .07, *P* = .002 - .02) than rural registration. Left-behind adolescents showed higher levels of risk-seeking, self- centeredness, simple-task (β = .05 – .07, *P* = .02 - .04) and lower levels of presence of MIL and family functioning (*β* = -0.05 – -0.07, *P* = .005 - .03).
